# A decade of multi-modality PET and MR imaging in abdominal oncology

**DOI:** 10.1259/bjr.20201351

**Published:** 2021-07-22

**Authors:** Lisa A. Min, Francesca Castagnoli, Wouter V. Vogel, Jisk P. Vellenga, Joost J.M. van Griethuysen, Max J. Lahaye, Monique Maas, Regina G.H. Beets Tan, Doenja M.J. Lambregts

**Affiliations:** 1 Department of Radiology, The Netherlands Cancer Institute, Amsterdam, The Netherlands; 2 GROW School for Oncology and Developmental Biology, University of Maastricht, Maastricht, The Netherlands; 3 Department of Radiology, University of Brescia, Brescia, Italy; 4 Department of Nuclear Medicine, The Netherlands Cancer Institute, Amsterdam, The Netherlands; 5 Department of Radiation Oncology, The Netherlands Cancer Institute, Amsterdam, The Netherlands; 6 Faculty or Health Sciences, University of Southern Denmark, Odense, Denmark

## Abstract

**Objectives:**

To investigate trends observed in a decade of published research on multimodality PET(/CT)+MR imaging in abdominal oncology, and to explore how these trends are reflected by the use of multimodality imaging performed at our institution.

**Methods:**

First, we performed a literature search (2009–2018) including all papers published on the multimodality combination of PET(/CT) and MRI in abdominal oncology. Retrieved papers were categorized according to a structured labelling system, including study design and outcome, cancer and lesion type under investigation and PET-tracer type. Results were analysed using descriptive statistics and evolutions over time were plotted graphically. Second, we performed a descriptive analysis of the numbers of MRI, PET/CT and multimodality PET/CT+MRI combinations (performed within *a* ≤14 days interval) performed during a similar time span at our institution.

**Results:**

Published research papers involving multimodality PET(/CT)+MRI combinations showed an impressive increase in numbers, both for retrospective combinations of PET/CT and MRI, as well as hybrid PET/MRI. Main areas of research included new PET-tracers, visual PET(/CT)+MRI assessment for staging, and (semi-)quantitative analysis of PET-parameters compared to or combined with MRI-parameters as predictive biomarkers. In line with literature, we also observed a vast increase in numbers of multimodality PET/CT+MRI imaging in our institutional data.

**Conclusions:**

The tremendous increase in published literature on multimodality imaging, reflected by our institutional data, shows the continuously growing interest in comprehensive multivariable imaging evaluations to guide oncological practice.

**Advances in knowledge:**

The role of multimodality imaging in oncology is rapidly evolving. This paper summarizes the main applications and recent developments in multimodality imaging, with a specific focus on the combination of PET+MRI in abdominal oncology.

## Introduction

Multimodality imaging in the context of diagnostic medical imaging can be defined as “the use of a combination of imaging techniques or platforms encompassing aspects of anatomical, functional or molecular imaging methods”,^1^ and it is often used in clinical practice as a term to describe the use of different imaging modalities to address a single medical problem. In oncology, multimodality imaging can aid in diagnosis, staging and treatment response monitoring by visualizing different tumour properties, thereby providing complementary information on both morphology and physiology. Different imaging modalities can either be combined retrospectively, after separate acquisition (with or without retrospective image registration and/or fusion), or by simultaneous acquisition (commonly referred to as “hybrid” imaging), of which PET/CT and the more recently introduced hybrid PET/MRI systems are the most familiar examples.

Advantages of “hybrid” acquisition include – apart from patient convenience – improved image co-registration and better opportunities to study and correlate dynamic disease processes *in vivo*, such as perfusion and tracer distribution, and tumour response to pharmacological and interventional treatments.^2,3^ PET/CT has already proven to be a valuable tool in the staging of a wide range of malignancies, and its use is recommended in many oncological guidelines.^4–9^ Owing to the growing array of tumour-targeted tracers, including prostate cancer radiotracers and tracers for somatostatin receptor imaging in neuroendocrine tumours, its clinical role keeps evolving.^10–13^

Already before the development of hybrid imaging systems, it was recognized that a multimodality combination of PET with anatomical imaging has many potential advantages. Combining PET with MRI offers the specific benefits of the superior soft-tissue contrast and image resolution of MRI, allowing detailed anatomical correlation and local staging.^14^ In addition, it allows multiparametric evaluations by combining the metabolic information from PET with functional MR sequences such as diffusion-weighted imaging (DWI) and dynamic contrast-enhanced (DCE) MRI, to allow simultaneous assessment of biological tumour properties such as metabolism, cellularity and perfusion. From a safety perspective, the lack of radiation in MRI is an additional property that makes MRI an attractive modality for repeated longitudinal follow-up and for paediatric imaging. The arrival of the first hybrid PET/MRI systems has further boosted the field of multimodality PET+MRI imaging and research.

With this paper, we set out to investigate trends in published research on multimodality imaging during the time span of a decade, with a specific focus on the combination of PET(/CT) and MRI in abdominal oncology. Second, we explored how trends observed in literature are reflected by the use of multimodality imaging at our own comprehensive European Cancer Centre.

## Methods and materials

### Literature search

A search strategy was constructed in PubMed (NCBI) to retrieve all English-language original research publications (2008–2018) combining PET/CT and MRI in a multimodality study setting, either acquired as stand-alone modalities (with or without retrospective image registration and/or fusion), or using bed system combined or fully hybrid PET/MRI systems. The search was restricted to studies focusing on abdominal oncology. Main search terms included “PET” and “MRI” and “abdominal malignancy” as well as terms referring to various abdominal regions, individual organs and specific tumour types (or their respective synonyms/MeSH-terms) in the title and/or abstract. Animal studies were excluded. Further details of the search strategy are provided in Uncited Supplementary Table 1 . All retrieved articles were reviewed by a single reviewer (LAM or FC), based on title and abstract, to assess eligibility for inclusion. In case of doubt, the other reader was consulted to reach consensus. Each included paper was labelled (using the Rayyan QCRI online application)^15^ according to the following descriptors:

1. Study design: prospective/retrospective, single-centre/multicentre, combination/correlation/comparison of PET and MRI:
  (note: combination = assessing complementary value of PET combined with MRI to predict a clinical outcome; correlation = assessing correlation between PET and MRI parameters (*e.g.* SUV and ADC), comparison = comparing diagnostic performance of PET to that of MRI);
2. Method of multimodality imaging: retrospective combination of stand-alone PET/CT and MRI with or without retrospective image fusion, bed system-combined PET/MRI, hybrid PET/MRI;
3. Type of PET-tracer(s);
4. Method of image evaluation: visual/qualitative, quantitative, other;
5. Study aim: lesion detection, correlation of PET and MRI parameters, response assessment, technical (*e.g.* sequence development and testing), prognostic (*e.g.* survival prediction), or other;
6. Cancer type;
7. Lesion type: primary tumour, nodes, metastases, mixed;

Uncited Supplementary Table 1.

### Analysis of literature data

Based on the assigned labels, annual numbers of research papers in each category and subcategory were determined and relative proportions (%) and cumulative effects over time were calculated using descriptive analyses in Microsoft Excel (Microsoft Office 2019, version 16.16.22, Redmond, WA, USA). Trends over time were plotted using Microsoft Excel and GraphPad Prism (GraphPad Software, version 7.03, San Diego, CA, USA).

### Institutional data

Our institute‘s internal picture archiving and communication system (PACS; Carestream Vue, version 11.4.1.1102, Carestream Health, Rochester, New York, USA) was searched for all MRI and PET/CT studies performed from 2008 to 2017 as part of routine clinical care. Patients who underwent a multimodality combination of both PET/CT and MRI within the same diagnostic workup (arbitrarily defined as studies performed within a time-interval of ≤14 days) were documented separately. For each individual study, the exam date, modality, PET-tracer used (if applicable), study description (*i.e.* body part and protocol) and pseudonymized patient identification number were stored. Studies were excluded if they were imported from another hospital or performed solely for protocol optimization (*e.g.* phantom studies, calibration series) or interventional guidance (*e.g.* MR-guided biopsy). Annual numbers of MRI, PET/CT and multimodality combinations of MRI+PET/CT were determined, and the relative increase over time compared to the baseline year was calculated and plotted in GraphPad.

## Results

### Main study characteristics

**Figure 1.**
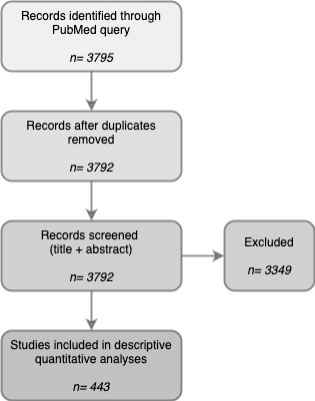
Literature selection process

**Figure 2.**
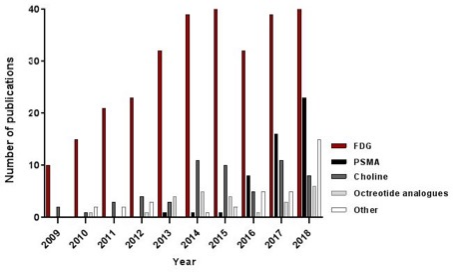
Evolution in the annual numbers of PET studies inpublished multimodality imaging research, specified for the PET-tracer(s) used. FDG: 18F-fluorodeoxyglucose; PSMA: prostate-specific membrane antigen; octreotide analogues: 68Ga-labelled somatostatin receptor ligands; ‘Other tracers’ includes tracers used in a single or few of the retrieved studies (*e.g.* fluciclovine, fluorothymidine (18F-FLT), fluoromisonidazole (18F-FMISO), dihydroxyphenylalanine (18F-DOPA)).

The literature selection process is illustrated schematically in the PRISMA flowchart in Figure 1. A total of 443 original research papers combining PET/CT and MRI in a multimodality study setting for abdominal malignancies were retrieved, including a total number of 60,725 patients. The PET-tracer used was 18F-labeled glucose analogue fluorodeoxyglucose ([^18^F] FDG, or “FDG”) in 294/443 studies, 149 studies used other non-FDG tracers (a combination of both FDG and non-FDG tracers was used in 14 studies). Trends over time are shown in Figure 2. Table 1 summarizes the detailed study characteristics for the main group of 294 FDG-PET(/CT)+MRI papers. The majority of these papers (211/294, 72%) retrospectively combined or compared FDG-PET/CT and MRI that were acquired separately, the remaining studies (28%) concerned combined PET/MRI acquisitions using either hybrid or bed system-combined PET/MRI scanners. Visual image assessment was the most commonly employed method of image evaluation (144/294, 49%), followed by papers focusing on quantitative imaging evaluation (96/294, 33%). The most frequently studied tumour types were gynaecological and colorectal cancer. The largest subgroups of papers focused on assessing the complementary value of PET(/CT) combined with MRI (127/294, 43%) or on comparing the diagnostic (or predictive) value of PET/CT to that of MRI (113/294, 38%).

**Table 1.** Summary of papers on multimodality assessment of FDG-PET and MRI in abdominal oncology

		Number	%
**Total**		294	100

**Study design**	Prospective	148	50
	Retrospective	134	46
	Unspecified	12	4
			
	Single-centre	281	96
	Multicentre	8	3
	Unspecified	5	2

	*Combination* of FDG-PET(/CT)+MRI (complementary value)	127	43
	*Comparison* of FDG-PET(/CT) *vs*MRI	113	38
	*Correlation* of FDG-PET(/CT) and MRI parameters	32	11
	Other	22	7

**Type of multimodality imaging acquisition**	Stand-alone (separate) acquisition of PET/CT and MRI	211	72
	*Without image fusion*	189	64
	*With retrospective image fusion*	22	7
	Hybrid PET/MRI acquisition	72	24
	Bed-system combined PET/MRI acquisition	11	4

**Method of image evaluation**	Visual (qualitative) assessment	144	49
	Quantitative assessment	96	33
	Technical (*e.g.* development and testing)	38	13
	Other	16	5

**Study aim**	Lesion detection	138	47
	Correlation between FDG-PET(/CT) and MRI parameters	46	16
	Response assessment and prediction	43	15
	Technical (*e.g.* sequence development and testing)	39	13
	Prediction of prognostic outcomes (*e.g.* survival)	20	7
	Other	8	3

**Tumour type**	Gynaecological	94	32
	Colorectal	63	21
	Mixed types	60	20
	Liver (primary + metastatic)	20	7
	Pancreas	20	7
	Upper GI (oesophagus, stomach)	12	4
	Urological (prostate, bladder, kidney)	11	4
	Anal	6	2
	Other (GIST, NET, adrenal, screening/volunteers)	9	3

**Lesion type**	Mixed	123	42
	Primary tumour	107	36
	Distant metastases	43	15
	Lymph nodes	21	7

### Evolution of PET-tracers used in multimodality imaging studies

As shown in Figure 2, FDG was the most frequently reported PET tracer (66%). Other reported tracers included mainly those used for prostate cancer imaging, that is, choline tracers (11C- or 18F-labelled phospholipid precursor)^16,17^ or prostate-specific membrane antigen (PSMA)-based tracers (68Ga- or 18F-labelled small-molecule ligands),^18–20^ and octreotide-based tracers (68Ga-labelled octreotide analogs targeted at the somatostatin-receptor, overexpressed in many neuro-endocrine tumours).^21–25^ After some incidental reports (<10/year) in the first half of the study period, reports on the use of these tumour-specific tracers showed a marked increase during the second half of the study period, with non-FDG tracers constituting a majority (55%) of the total number of multimodality imaging research reports in 2018, the final study year.

### Evolutions in stand-alone versus hybrid PET/MRI studies

**Figure 3.**
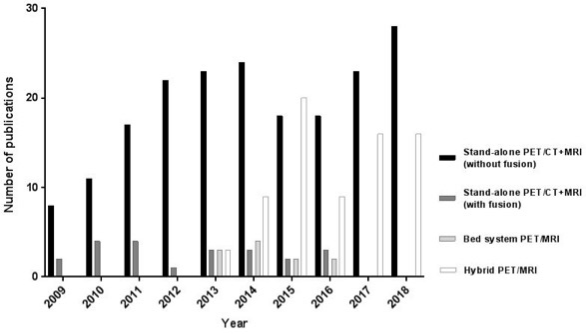
Evolution in the annual numbers of original research publications on multimodality combinations of FDG-PET/CT+MRI or PET/MRI in abdominal oncology specified per acquisition approach, *i.e*. retrospective combination of separately acquired FDG-PET/CT and MRI (with or without retrospective image fusion) versus prospective combination of PET and MRI using either bed-system combined acquisition or fully hybrid acquisition.

Figure 3 compares the evolution of research focusing on retrospective combinations of FDG-PET/CT and MRI, versus prospectively combined FDG-PET/MRI acquisition studies. Of the 211 studies that retrospectively combined FDG-PET/CT and MRI, only a small minority or early studies applied image fusion (22/211, 10%). After the introduction of the first commercially available hybrid PET/MRI scanners in 2011, studies with hybrid PET/MRI started appearing in 2013. There was a steady increase in the following years and a striking peak in 2015, when the number of hybrid FDG-PET/MRI studies even exceeded the number of retrospectively combined multimodality PET/MRI studies. Studies using bed system-combined PET/MRI scans (where the patient is moved between a separate PET/CT and MRI scanner on a single bed, for direct sequential scanning without the need of patient repositioning) were sparse (11/294, 4%), and for this review (focusing on abdominal oncology), the last retrieved report of this system dates from 2016.

### Image evaluation approaches

**Figure 4.**
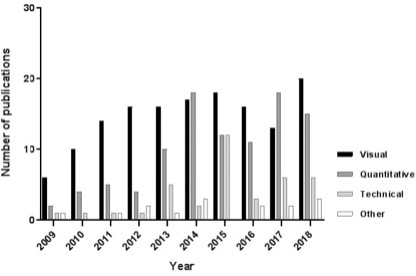
Evolution in the annual numbers of original research publications on multimodality combinations of FDG-PET/CT+MRI inabdominal oncology, specified per image evaluation approach, *i.e.* visual (qualitative) assessment, quantitative assessment, technical studies (i.e. protocol optimization and testing) and “other” (*e.g.* delineation studies for radiotherapy planning).

As shown in Figure 4, approximately half of the papers combining FDG-PET/CT and MRI (144/294, 49%) focused on visual (qualitative) image assessment (mainly lesion detection for primary tumour staging), with more or less consistent numbers of reports over time. The main tumour types under investigation are detailed in Table 2 and included gynaecological and colorectal cancers. A considerable increase over time was observed for studies applying quantitative methods of imaging assessment, including measurements such as the standardized uptake value (SUV, from PET), apparent diffusion coefficient (ADC, the main quantitative measure of DWI), parameters from dynamic contrast-enhanced MRI (*e.g.* Ktrans), and volumetric measurements. These quantitative studies constituted 33% of the total cohort, and mainly focused on correlation between FDG-PET and MRI parameters or on use of these parameters as “biomarkers” to predict clinical outcomes. Table 3 summarizes the main findings of this latter subgroup of papers focusing on FDG-PET(/CT) and MRI parameters used as biomarkers to predict response and/or survival, the two most investigated clinical outcomes.

**Table 2.** Summary of papers focusing on multimodality combination of PET and MRI for visual lesion detection (for tumour staging)

Tumour type	Total no. of studies (%)	Median number of patients per study (range)
Tumour types/groups with ≥ 10 available studies		
Gynaecological cancers	43 (36)	43 (12–493)
*Retrospective combination (separate acquisition*)	*34 (28*)	*51.5 (12–493*)
*Combined acquisition (hybrid or bed-system PET/MRI*)	*9* (*8*)	*27 (18–71*)
Colorectal cancer	32 (27)	34.5 (12–352)
*Retrospective combination (separate acquisition*)	*27 (23*)	*35 (18–352*)
*Combined acquisition (hybrid or bed-system PET/MRI*)	*5* (*4*)	*26 (12–55*)
Mixed tumour types	15 (12)	37 (15–237)
*Retrospective combination (separate acquisition*)	*10 (8*)	*45.5 (15–237*)
*Combined acquisition (hybrid or bed-system PET/MRI*)	*5* (*4*)	*66 (32–173)*
Tumour types/groups with ≤10 available studies		
Pancreas	10 (8)	48 (27–644)
Urological (prostate, bladder, kidney)	6 (5)	55 (22–287)
Anal	5 (4)	43 (11–61)
Upper GI (oesophagus, stomach)	4 (3)	46 (19–49)
Liver	3 (3)	35 (12–111)
Other (GIST, adrenal)	2 (2)	12.5 (9–16)

**Table 3.** Overview of papers focusing on multimodality combination of PET and MRI for prediction of treatment response and/or survival, based on (semi-)quantitative image parameters from imaging.

Study	n=	Tumour type (+lesion type)	Imaging modalities	Clinical outcome(+outcome definition)	Key findings	Added value of combining PET and MRI?	Combination with non-imaging (clinical) predictors?	Comments
Gynaecological malignancies								
Bowen *et al*. (2018)^26^	21	cervix (primary tumour)	PET/CT, DWI, DCE-MRI	Response (tumour volume < vs. ³10% of baseline measured 1 month post-treatment)	Predictors of response: pre-therapy SUVmean (AUC 0.81) & SUVmax (AUC 0.81) after 2 weeks of treatmemt: ΔADCskewness (AUC 0.86) after 5 weeks of treatment: ADCmean (AUC 0.81), %ΔSUVmean (AUC 0.79), ΔSUVskewness (AUC 0.79)	Not reported	No	Univariable ROC analysis.
Lucia *et al*. (2018)^27^	102	cervix (primary tumour)	PET/CT, T2W, DWI, DCE-MRI	Survival & local control (DFS; locoregional control)	DFS predictors: ADC Entropy_GLCM_-Q_F_ ≤ 12.64 (HR: 30.95), CE-MRI, RLVAR_GLRLM_-Q_L_ ≤ 0.17 (HR: 11.33); Locoregional control independent predictors: ADC Entropy_GLCM_-Q_F_ ≤ 12.64 (HR: 16.35), PET GLNU_GLRLM_-Q_E_ ≤ 103.71 (HR: 20.01)	Yes	Yes (age, FIGO, N-stage, BMI, blood cell counts, RTx dose, treatment time)	Uni- & multivariable survival analysis, independent training and testing cohorts
Sarabhai *et al*. (2018)^28^	8	cervix (primary tumour)	PET/MRI with DWI and DCE-MRI	Response (RECIST + PERCIST CR/PR *vs* SD/PD measured 2–6 wk after treatment)	Predictors of response: mean Δtumour size −60%, ΔSUVmax −64%, ΔSUVmean −62%, ΔADCmin + 38%, ΔADCmean + 39%, ΔKtrans −39%, ΔKep −47%, ΔiAUC −57%	Not reported	No	Heterogeneous histology and treatments. Descriptive analysis only, only one non-responder.
Rahman *et al*. (2016)^29^	90	cervix (primary + nodes)	PET/CT, T2W	Survival (PFS; OS)	PFS predictors: SUVmax ≤ 10.7 (HR: 2.87) and MTV ≤ 26.5 (HR: 7.58) or TLG ≤ 231 (HR: 4.54) in scc; SUVmax ≤ 13.4 (HR: 12.9) in nscc; OS predictors: MTV ≤ 30.4 (HR: 10.6) or TLG ≤ 231 (HR 11.6) in scc; SUVmax ≤ 14.1 (HR: 6.98) in nscc	No	Yes (age, FIGO, *N* + stage, surgery)	Uni- and multivariable survival analysis. Results stratified for scc *vs* nscc histology.
Ho *et al*. (2017)^30^	69	cervix (primary tumour)	PET/CT, DWI	Survival (DFS; OS; central/locoregional/distant recurrence free survival (RFS))	DFS predictors: ADCmean (>0.940×10^−3^; HR: 0.36), FIGO-stage I/II (HR: 2.4), nscc (HR: 0.23); OS, central RFS and locoregional RFS: no significant predictors; - Distant RFS predictor: nscc (HR: 0.12)	No	Yes (age, FIGO, histology scc/ncc, differentiation grade, N0 *vs N* + disease)	Uni- & multivariable survival analysis.
Ueno *et al*. (2017)^31^	21	cervix (primary tumour)	PET/CT, DWI	Response & survival (RECIST/PERCIST CR/PR *vs* SD/PD; event-free survival (EFS))	Predictors of response: TLG (AUC: 0.84, optimal cut-off ≥ 679.69 g), MTV (AUC: 0.78, optimal cut-off ≥ 71.47 ml); Predictors of impaired EFS: MTV ≥ 71.47 ml (HR: 4.73), TLG ≥ 679,69 g (HR: 4,73), ADC10% ≥ 0.86×10^−3^ mm^2^/s (HR: 5,21)	Yes	No	Response: univariable ROC analysis; EFS: uni- & multivariable survival analysis.
Micco *et al*. (2014)^32^	49	cervix (primary tumour)	PET/CT, DWI, DCE-MRI	Survival (DFS; OS)	DFS predictors: FIGO-stage IB/IIA (HR: 3.89), LN-neg (HR 6.15), max. tumour diameter (HR: 1.47), ADCmean (HR: 1.56), MTV (HR: 1.31), TLG (HR: 1.03) OS predictors: FIGO-stage IB/IIA (HR: 6.45), LN-neg (HR: 7.8), ADCmean (HR: 0.46), MTV (HR: 1.42)	Not reported	Yes (FIGO, N-stage, histology scc/nscc, grade, tumour size)	Univariable survival analysis.
Nakamura *et al*. (2014)^33^	80	cervix (lymph nodes)	PET/CT, DWI	Survival (DFS; OS)	DFS predictors: LN SUVmax ≤ 2.10 (HR: 6.65); OS predictors: LN SUVmax ≤ 2.225 (HR: 3.05)	No	No	Univariable ROC analysis, uni- & multivariable survival analysis.
Nakamura *et al*. (2012)^34^	66	cervix (primary tumour)	PET/CT, DWI	Survival (DFS; OS)	DFS predictors: FIGO-stage IB/IIA (HR: 5.265), LN-neg (HR: 4.124), SUVmax ≤ 15.55+ADCmin ³0.61 (HR: 8.779); OS predictors: FIGO-stage IB/IIA (HR: 11.922), LN-neg (HR: 8.659), SUVmax ≤ 15.55+ADCmin ³0.61 (HR: 8.449)	Yes	Yes (FIGO, pelvic *N* + disease, histology scc/nscc, tumour size)	Uni- & multivariable survival analysis.
Nakamura *et al*. (2013)^35^	131	endometrium (primary tumour)	PET/CT, DWI	Survival (DFS; OS)	DFS predictors: FIGO-stage I/II (HR: 11.49), SUVmax ≤ 17.70 (HR: 13.33); OS predictors: FIGO stage I/II (HR: 15.15), SUVmax ≤ 18.42 (HR: 15.63)	No	Yes (age, FIGO, histology, N-stage, lymhopvascular invasion, ovarian M+, peritoneal cytology)	Univariable ROC analysis, Uni- & multivariable survival analysis.
Rectal cancer								
Joye *et al*. (2017)^36^	85	rectum (primary tumour)	PET/CT, T2W, DWI	Response (yPT0-1N0 *vs* other yPTN)	Predictors in optimal model: SUVpeak post-CRT, ADC post-CRT, ADC ratio pre-CRT/post- CRT, diameter sphere post-CRT, Δ%diameter sphere post-CRT (0.46). Model AUC 0.83, sensitivity: 75%; specificity 94%	Yes	Yes (cytokines, gene expression profiles)	Multivariable analysis; cross-validated.
Nishimura *et al*. (2016)^37^	15	rectum (primary tumour)	PET/CT, T2W	Response (TRG1-2 *vs* TRG3)	Significant results: Responders on MRI: smaller tumour size post-CRT, larger decrease in size post-CRT Responders on PET: lower SUVmax during and post-CRT, larger decrease in SUVmax during and after CRT	Not reported	Yes (age, sex, tumour size, chemotherapy regimen, histology)	Fishers exact test.
Heijmen *et al*. (2015)^38^	39	rectum (liver metastasis)	PET/CT, DWI, T2*	Survival and response (PFS; OS; size change)	PFS predictors: pre-chemo ADCmean (HR: 0.749/0.1×10^–3^ mm^2^/s); OS predictors: pre-chemo SUVmax (HR: 1.125), TLG (HR: 1.047/100g), and ADCmean (HR 0.667/0.1×10^–3^ mm^2^/s); T2* (HR: 1.118/ms); No significant predictors for response	Yes, but effect not specified	No	Univariable survival analysis. (No detailed results for multivariable and response analysis).
Ippolito *et al*. (2015)^39^	31	rectum (primary tumour)	PET/CT, DWI	Response (TRG1-2 *vs* TRG3-5)	Predictors of response: SUVmax post-CRT (AUC: 0.889, optimal cut-off: 4.4), ADCmean post-CRT (AUC: 0.815, optimal cut-off: 1.294 10^−3^ mm^2^/s)	Not reported	No	Univariable ROC analysis.
Ippolito *et al*. (2012)^40^	30	rectum (primary tumour)	PET/CT, DWI	Response (TRG1-2 *vs* TRG3-5)	Predictors of response: SUVmax post-CRT < 4.4, ADCmean post-CRT > 1.294×10^−3^ mm^2^/s	Yes, but effect not specified	No	Univariable regression analysis. (No detailed results for multivariable analysis)
Herrmann *et al*. (2011)^41^	28	rectum (primary tumour)	PET/CT, T2W	Response (<10% residual tumour cells *vs* ≥ 10%)	Predictors of response, during CRT: Δ%SUVmean (AUC: 0.70–0.75); Predictors of response, post-CRT: Δ%SUVmean (AUC: 0.75–0.76), Δ%PETvolume (AUC: 0.73–0.76),	Not reported	No	Univariable ROC analysis.
Lambrecht *et al*. (2010)^42^	22	rectum (primary tumour)	PET/CT, DWI	Response (pCR *vs* non-pCR)	Pre-CRT predictors: ADCmean (<1.06×10^−3^ mm^2^/s, sens: 1.0, spec: 0.88) During CRT predictors: Δ%SUVmax (>-40%, sens: 1.0, spec: 0.75), ADCmean pre- CRT < 1.06×10^−3^ mm^2^/s + Δ%SUVmax during CRT >-40% (sens: 1.0, spec: 0.94) Post-CRT predictors: Δ%SUVmax (>-76%, sens: 1.0, spec: 0.75), ADCmean pre-CRT < 1.06+Δ%SUVmax post-CRT >-76% (sens: 1.0, spec: 1.0), Δ%SUVmax during CRT >-40% + Δ%SUVmax post-CRT >-76% (sens: 1.0, spec: 0.94)	Yes	No	Univariable ROC analysis.
Other tumour types								
Fang *et al*. (2018)^43^	20	oesophagus (primary tumour)	PET/CT, DWI	Response (TRG1 *vs* TRG2-5)	Predictors of response during CRT: Δ%ADCmean (AUC: 1.0), Δ%ADCmedian (AUC: 0.99), Δ%ADC10% (AUC: 1.0), Δ%ADC25% (AUC: 1.0), Δ%ADC75% (AUC: 0.97), Δ%TLG (AUC: 0.95) No predictors of response pre- and post-CRT	Not reported	No	Univariable ROC analysis.
Lee *et al*. (2016)^44^	11	stomach (primary tumour)	PET/MRI with DWI and DCE-MRI	Response (RECIST CR + PR vs. SD + PD)	Predictors of response: Ktrans mean (AUC: 0.917), iAUC mean (AUC: 0.867)	No	No	Univariable ROC analysis.
Weber *et al*. (2013)^45^	15	oesophagus and oesophagogastric (primary tumour)	PET/CT, DWI	Response (PET response; clinical response *vs* non-response; histopathological regression Grade 1 + 2 vs. Grade 3)	Significant results: PET response: larger Δ%ADCmean and Δ%SUVmean during chemo Clinical response: no significant results Histopathological response: higher ADCmean pre-chemo in Grade 1 + 2	No	No	Student’s T-test.
Hong *et al*. (2017)^46^	52	HCC (primary tumour)	PET/CT, DWI	Survival (Disease Specific Survival (DSS))	Predictors of impaired DSS: SUVmax tumour/SUVmean normal liver ≥ 2 (HR: 2.46), T-stage (HR: 3.01), PIVKA-II ≥ 100 mAU/ml (HR: 5.11), surgery as initial treatment (HR: 0.04)	No	Yes (age, sex, Edmondson grade, Child-Pugh, MELD score, AFP, PIVKA-II, lesion n^o^, T-stage, surgery)	Multivariable survival analysis. Cut-offs based on literature.
Han *et al*. (2014)^47^	298	HCC (primary tumour)	PET/CT, CE-MRI	Survival (clinical + radiological recurrence; OS)	Recurrence predictors: SUV > 3.5 (HR: 2.025), male (HR: 2.192), AFP > 100 ng ml^−1^ (HR: 1.888); Impaired OS predictors: SUV > 3.5 (HR:7.331), AFP > 100 ng ml^−1^ (HR: 3.061)	No	Yes (age, sex, platelets, bilirubin, Indocyanin green, Child-Pugh, MELD, AFP, PIVKA-II, lesion size/n^o^)	Multivariable survival analysis.
Chen *et al*. (2018)^48^	63	pancreas (primary tumour)	PET/MRI with DWI, DCE-MRI and MR spectroscopy	Survival (OS, time to progression (TTP))	OS predictors: TLG/peak (<11.81, HR: 4.610), ADCmin (>0.844×10^−3^ mm^2^/s, HR: 0.999); TTP predictors: TLG/peak (<11.81, HR: 2.130), TLG (<33 g, HR: 1.004)	Yes	Yes (age, sex, TNM-stage)	Multivariate survival analysis.
Wang *et al*. (2018)^49^	13	pancreas (primary + metastasis)	PET/MRI with DWI	Response & survival (PFS; OS; RECIST PR *vs* SD + PD)	Predictors of response during chemo: Δ%MTV (≥−60%, AUC: 0.95), Δ%TLG (≥−65%, AUC: 0.95), Δ%ADCmean (≥+20%, AUC: 0.91), Δ%ADCmin (≥+20%, AUC: 0.86) Predictors of PFS and OS: Δ%MTV ≥−60%, %TLG ≥−65%, Δ%ADCmean ≥+ 20%	Not reported	No	Univariable ROC and survival analysis.
Chen *et al*. (2016)^50^	60	pancreas/periampullar (primary tumour)	PET/MRI with DWI, MR spectroscopy	Survival (PFS)	Predictors of impaired PFS: MTV/ADCmin ratio (HR: 1.036)	Yes	Yes (age, sex, tumour size, TNM-stage)	Multivariable survival analysis.

ADC, apparent diffusion coefficient (DWI); ADC Entropy_GLCM_-Q_F_, gray-level co-occurrence texture parameter from the ADC map; AFP, alpha-fetoprotein; CE-MRI RLVAR_GLRLM_-Q_L_, gray-level run-length matrix texture parameter from the contrast-enhanced MRI image; CR, complete response (RECIST); CRT, chemoradiotherapy; DCE-MRI, dynamic contrast-enhanced magnetic resonance imaging; DFS, disease-free survival; DSS, disease-specific survival; DWI, diffusion-weighted magnetic resonance imaging; EFS, event-free survival; FIGO, International Federation of Gynecology and Obstetrics; HCC, hepatocellular carcinoma; Kep, reverse reflux rate constant (DCE-MRI); Ktrans, volume transfer coefficient (DCE-MRI); LN, lymph node; MR, magnetic resonance; MTV, metabolic tumour volume (PET); OS, overall survival; PD, progressive disease (RECIST); PERCIST, PET response criteria in solid tumours; PET/CT, positron-emission tomography/computed tomography; PET GLNU_GLRLM_-Q_E_, gray-level run-length matrix texture parameter from the PET image; PFS, progression-free survival; PIVKA-II, prothrombin induced by vitamin K absence-II; PR, partial response (RECIST); RECIST, response evaluation criteria in solid tumours; RFS, recurrence-free survival; ROC, receiver operating curve; SCC, squamous cell carcinoma; SD, stable disease (RECIST); SUV, standardized uptake value (PET); T2*, susceptibility-weighted MRI; TLG, total lesion glycolysis (PET); TRG, tumour regression grade; TTP, time to progression; T2W, T2-weighted magnetic resonance imaging; chemo, chemotherapy;iAUC, initial (60 seconds) area under the gadolinium concentration curve (DCE-MRI); nscc, non-squamous cell carcinoma; pCR, pathological complete response; sens, sensitivity; spec, specificity; wk, weeks; yPT, pathological treatment response.

A minority (38/294, 13%) of reports concerned “technical” studies that describe the development, optimization and testing of new acquisition techniques. These studies showed a peak in the first years after the introduction of the first hybrid PET/MRI systems, and included mostly studies on MRI-based attenuation correction techniques^51–57^ and quality of image co-registration.^58–65^ There was a final small subgroup (16/294, 5%) of “other” studies, which for example included delineation studies (for radiotherapy planning).^66,67^

### Institutional data

**Figure 5.**
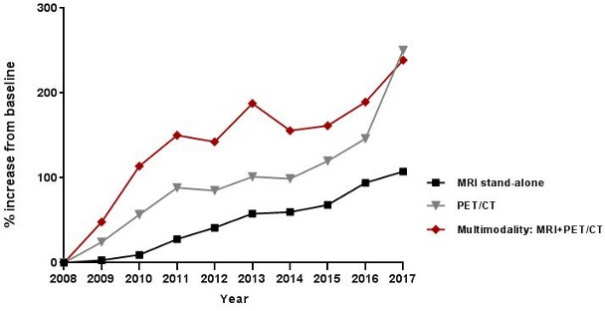
Annual growth of MR imaging studies, PET/CTs and multimodality MRI+PET/CT imaging combinations observed in our institution relative to the benchmark year 2008.

During the ten-year study interval, 53.537 MRIs, 27.003 PET/CTs and 5.660 multimodality MRI+PET/CT combinations (performed within *a* ≤14 day interval) were performed at our institution, of which the developments are shown in Figure 5 (Hybrid PET/MRI is not available at our institution). The overall ten-year increase relative to the baseline year (2008) was 108% for MRI, 250% for PET/CT and 239% for the multimodality combination of MRI+PET/CT, with consistently larger proportional growth of multimodality PET/CT+MRI combinations compared to either PET/CT or MRI on their own (with the exception of the final study year). The multimodality PET/CT+MRI combinations included 698 cases where PET/CT was combined with abdominal MRI examinations, and in line with our literature findings gynaecological and colorectal cancer were amongst the main tumour types under investigation.

## Discussion

Aim of this paper was to describe main evolutions observed in a decade of published research on multimodality MRI and PET(/CT) imaging in abdominal oncology, and to see how these trends are reflected in data from our own institution. Annual numbers of published PET(/CT)+MRI research (as well as PET/CT+MRI combination studies performed at our own institution) showed a gradual and vast increase over time, with gynaecological and colorectal cancer being amongst the main tumour types under investigation. A major boost in PET(/CT)+MRI research was observed after the introduction of the first hybrid PET/MRI systems, which fully replaced earlier data on retrospective image fusion and bed-system combined (sequential) PET/MRI. Although a main focus of research throughout the study period remained combined use of PET/CT and MRI for visual diagnostic evaluations (*i.e.* lesion detection and tumour staging), quantitative analysis of PET- and MRI-based parameters as biomarkers of disease took flight in the second half of the study period. Another major development was the increased use of more tumour-specific tracers (other than FDG) in multimodality imaging, in specific the combination of PSMA-based PET(/CT) and MRI in prostate cancer.

### Stand-alone versus hybrid combination of PET and MRI

The majority (72%) of studies retrieved by our literature search concerned FDG-PET/CT and MRI examinations acquired sequentially, that is, as stand-alone modalities. The largest subgroup of these reports (65%) were studies that compared the diagnostic value of FDG-PET/CT to that of MRI, but a significant proportion (33%) evaluated the complementary value of combining FDG-PET/CT with MRI, which are essentially the studies that fall within the scope of our current paper focusing on “multimodality imaging”. In our instutional analysis, a remarkable increase was also observed during the study period in the number of multimodality PET/CT + MRI combinations performed as part of the same diagnostic work up. These findings suggest that PET and MRI offer complementary information (both anatomical and functional) that is of growing relevance for diagnostic oncologic imaging evaluations. This notion likely also led to the development of hybrid PET/MRI systems that became commercially available in 2011. Their introduction gave rise to a quickly growing number of hybrid PET/MRI reports in literature during the direct following years, including a peak in technical reports (*e.g.* on MR-based attenuation correction techniques and image co-registration) during the early study years up to 2015. In the same period, published research applying retrospective image fusion of separately acquired FDG-PET/CT and MRI, as well as bed-system combined sequential MRI acquisition more or less disappeared, which is likely related to competition of these techniques with the newly available and logistically more attractive hybrid image acquisition techniques.

Although hybrid PET/MRI is considered by many to be the next state-of-the-art image modality in oncological research, its implementation is still an ongoing process that is to date mostly limited to a number of expert clinics and specialized oncological and/or dedicated research centres. Initial reasons for scepticism included concerns about the image quality as a result of technical adaptations required for PET and MR integration, and the substantially higher costs for installation and operation of these devices. Defining the clinical and research areas where there is a specific benefit of hybrid PET/MRI acquisition also remains a topic of debate. Currently, there seems to be agreement that the value of hybrid PET/MRI lies mainly in comprehensive regional evaluation of the local tumour and its direct (micro-)environment, rather than competing with PET/CT for whole-body applications.^3,68^ In a recent scoping review, Morsing et al concluded that preliminary data suggest a superiority of PET/MRI for the detection of local recurrence in prostate cancer, local tumour invasion in cervical cancer, and liver metastases in colorectal cancer.^69^ From the studies included in our literature study, it seems that overall the respective benefits of PET (*i.e.* staging of lymph nodes and distant metastases) and MRI (detailed local tumour staging) are maintained with simultaneous PET/MRI acquisition,^70–72^ with the added benefit of improved imaging efficiency and potentially increased staging confidence.^2,14,73,74^ There have, however, so far been no studies that directly compared hybrid PET/MRI to separately acquired PET(/CT) and MRI to validate these effects. Other emerging and more unique applications of hybrid PET/MRI acquisition include theranostic imaging^75^ and *in vivo* dynamic evaluation of tumour biology, early tumour response and tracer kinetics, but these applications are still in early stages of research with only limited (pilot) data available.^76,77^

### PET-tracers

Another major development observed during the study period was the increased use of non-FDG, more tumour-specific PET-tracers, as illustrated in Figure 2, with studies using non-FDG tracers constituting even the majority of reports in the final study year. This disproportionate increase probably reflects some publication bias where results of novel tracer types – particularly positive results – are more likely to be published. Prostate-specific membrane antigen (PSMA)-targeted and choline tracers used in prostate cancer imaging, and octreotide analogues that target the somatostatin receptor often overexpressed by neuro-endocrine tumours, were the most frequently reported. Their value lies primarily in the detection of lymph nodes and distant metastases from these specific malignancies that typically exhibit a heterogeneous or low glucose metabolism and are, therefore, less susceptible to detection by FDG-PET. Recent guideline updates have embraced the use of these novel tracers. For example in prostate cancer, PSMA-PET (or alternatively choline-PET) is now recommended for patients with biochemical recurrence who are considered for salvage treatment,^6^ with growing evidence that PSMA-PET is superior to choline-PET for this purpose.^78^ For primary staging of prostate cancer, PET is currently not recommended by the guidelines, but evidence that PSMA-PET/MRI may also be beneficial for these indications is emerging.^79,80^

### Complementary value of FDG-PET and MRI for lesion detection and tumour staging

Despite abovementioned recent advances in tumour-specific tracers, 18F-FDG remains the main workhorse used for multimodality PET(/CT)+MRI imaging in oncology. The abdominal tumour types most often assessed with FDG-PET(/CT) and MRI within our literature study (as well as in our institutional data) were gynaecological and colorectal cancers, which accounted for 32 and 21% of all studies. As summarized in Table 2, studies focusing on lesion detection and staging varied considerably in terms of patient numbers and use of retrospective versus hybrid combinations of FDG-PET and MRI. For the gynaecological group, most evidence is based on studies involving cervical cancer patients, with the largest study including a cohort of 493 patients. In this study, Kim et al^81^ constructed and validated a nomogram to predict lymph-node metastasis in patients with early stages of cervical cancer, which included tumour size on MRI, suspicion of lymph node metastasis on whole-body FDG-PET/CT and patient age as independent predictors, resulting in a model performance of AUC 0.825 (95% CI 0.736–0.895) in the validation set. An earlier study already showed that fused FDG-PET and MRI images resulted in higher accuracy for detection of lymph node metastasis than FDG-PET/CT only (AUC 0.735 *vs* 0.690; *p* = 0.045) in a cohort of 79 patients with FIGO stage Ib-IVa cervical cancer, again suggesting added value for the combination of PET and MRI in this setting.^82^ Sarabhai et al^70^ compared hybrid PET/MRI with only the MRI component, and found an improvement in diagnostic accuracy for PET/MRI. Not surprisingly, this benefit involved lymph node metastasis (accuracy 87% *vs* 77%) and distant metastasis (accuracy 91% *vs* 83%), but not local staging (85% *vs* 87% correct T-stage). Also for recurrent gynaecological malignancies, hybrid PET/MRI was shown to outperform diagnostic accuracy of the whole-body MRI component alone.^83^ Combined use of MRI (for local staging) and PET/CT (for distant staging) has been adopted as a recommended strategy in the most recent joined guidelines on cervical cancer from the European Society of Gynaecological Oncology (ESGO), the European Society for Radiotherapy and Oncology (ESTRO), and the European Society of Pathology (ESP), in particular for patients considered for curative intent chemoradiotherapy. Use of hybrid PET/MRI as an alternative approach is not specifically mentioned or discussed.^84^

In colorectal cancer, MRI is routinely used for detailed local staging in rectal cancer and has a known added benefit compared to CT for the detection of liver metastases, in particular for small lesions.^85,86^ For primary staging in case of localized disease, PET/CT is not routinely recommended in current guidelines.^87^ PET/CT is mainly advised as a problem solver in addition to routine staging, for the detection of extra hepatic disease (in candidates for local treatment of liver metastasis) and for the detection of recurrent disease after primary resection.^88^ Vigano et al studied the role of FDG-PET/CT in 107 colorectal cancer patients before resection of liver metastasis. FDG-PET/CT revealed extra-hepatic disease (mainly lymph nodes and peritoneal disease) in 28.8% (17/56) of the cases, which prevented futile liver resection in 20.3% (15/74) of patients deemed resectable by CT and/or MRI.^89^ Use of PET is also increasingly being studied to assess response to chemotherapy or chemoradiotherapy in colorectal cancer and several studies have suggested a possible complementary role for FDG-PET/CT next to MRI for detection of a complete local response, detection of remaining pelvic lymph nodes and distant metastasis after treatment.^90–92^ Catalano et al^93^ were among the first to compare the (re-)staging accuracy of FDG-PET/CT and hybrid PET/MRI in colorectal cancer. In a small series of 26 patients, assigned stage was discordant between the two hybrid modalities in 7/26 patients, and all but one patient were correctly staged using PET/MRI. Further evidence on whether there is a potential benefit to perform hybrid PET/MRI in colorectal cancer is sparse.

Finally, there have been some reports in mixed abdominal cancer types suggesting that PET and MRI may have a complementary value to improve overall diagnostic staging confidence and for the diagnostic management of patients with peritoneal carcinomatosis. Wang et al^94^ studied 128 patients (including ±48% colorectal cancer patients) that were considered for cytoreductive surgery and hyperthermic intraperitoneal chemotherapy (HIPEC) and had undergone FDG-PET/CT, of which 91 in adjunct to CT and/or MRI. In the latter group, PET/CT had a complimentary role which contributed to patient management in 33/91 cases by confirming or excluding peritoneal and/or extraperitoneal disease. In a study combining FDG-PET/CT and MRI for side-by-side-diagnostic assessment of 201 patients with different abdominal cancer types, a net increase in diagnostic confidence was seen compared to separate assessment of either PET/CT or MRI, with potential clinical impact in 1 out of 9 study patients.^14^

### Quantitative studies on PET and MRI biomarkers

As shown in Figure 5, we observed a significant increase over time in published reports focusing on quantitative PET(/CT)+MRI assessment, eventually constituting approximately one third of all reports in the final year of our literature review. These studies look beyond lesion detection and regard the images as a dataset, which can be used to render quantifiable variables that may serve as biomarkers to predict clinical outcomes such as tumour stage, treatment outcome and survival^26,28,31,32,36–39,41,43–45,49,50^ or correlate with other prognostic tumour markers such as histological tumour grade, hypoxia or microvascular invasion.^95–99^ ADC and SUV were amongst the most frequently reported imaging markers, and several studies reported a significant inverse correlation between higher tumour SUV values and lower ADCs.^30,46,100–107^ The common hypothesis is that tumours with a high cellular density (that show restricted diffusion and therefore low ADC values) will typically also exhibit an increased glucose metabolism, reflected by high SUV values. summarizes the main findings of studies focusing on use of PET and MRI biomarkers to predict response and/or survival, which constituted the two main investigated clinical outcomes. Methodology and results of these studies were highly variable. Despite this variation, a recurring finding was that higher tumour SUV, MTV or TLG and lower ACD values are generally associated with unfavourable outcomes (incomplete response, disease recurrence, reduced survival). It is worth mentioning that many of the studies in are preliminary reports that compare, rather than combine, the value of PET- and MRI-derived variables as predictors in univariable analysis.^26,28,32,37,39,41–45,49^ Overall, there were fourteen studies (out of the 25 included in ) that combined PET and MRI- parameters as potential outcome predictors in more comprehensive multivariable analyses,^27,29,31,33–36,38,40,46–48,50,108^ of which 6/14 found complementary value for the two techniques.^27,31,34,36,48,50^ In the remaining eight reports, either no complementary value was found (6/14 studies) or this was not explicitly analysed or reported (2/14 studies). Only two reports included (cross-) validation of data.^27,36^

Amongst the papers with positive findings on the combined use of PET and MRI parameters, Joye et al developed a model incorporating PET and MRI, but also molecular variables, to predict response to chemoradiotherapy in rectal cancer. They found that combining the multimodality information from PET and MRI resulted in optimal predictive performance, outperforming prediction models based on either of the two imaging modalities on its own or those based on molecular markers.^36^ In a preliminary study including a total of 102 patients (training *n*= 69, testing *n*= 33), Lucia et al^27^ evaluated the value of 92 pre-therapy PET/CT and MRI (*T*_2_-weighted, DWI and DCE-MRI) texture parameters to predict locoregional control and disease-free survival in patients treated with chemoradiotherapy for locally-advanced cervical cancer. They found a Radiomics signature based on a combination of ADC (Entropy-GLCM) and PET (GLNU-GLRLM) parameters to be highly predictive for locoregional control (AUC 1.0). Additional large-scale research, preferably including independent validation cohorts, is required to help further establish the benefit of multimodality quantitative PET+MRI evaluation in building clinical models that predict outcome and prognosis.

Our study has some limitations. Firstly, the scope of this review, “multimodality PET/CT and MRI in abdominal oncology” is too wide (including a wide range of tumour types, study designs and studied outcomes) to provide an in-depth or systematic review of all available literature. Our primary aim was to provide (including a wide range of tumour types, study designs and studied outcomes) to provide an in-depth or systematic review of all available literature. Our primary aim was to provide a broad overview of observed trends and highlight some key developments. Secondly, our institutional data was retrieved as raw data from the PACS system, and the large numbers did not allow a detailed (per-patient) classification to be fully in line with the literature search. Our institutional data analysis was mainly intended to provide some insights into how trends observed in literature translate to evolutions in the use of multimodality imaging in an oncologic referral centre, using our institutional data as an anecdotal example.

## Conclusions

This review has shown that the field of multimodality imaging has evolved in several ways. During the study period hybrid PET/MRI systems were introduced, which gave rise to a major novel field of research, while at the same time shifting the focus away from retrospective PET(/CT)+MRI image fusion and bed system-combined PET/MRI acquisition. New PET-tracers have found their way into clinical practice. Studies focusing on combined quantitative analysis of PET and MRI data have taken flight and (multiparametric) predictive models incorporating these imaging biomarkers to predict clinical outcomes such as survival and treatment response are now being developed and tested. The next decade of research will need to further establish the true clinical potential of such prediction tools as well as define the definite role of hybrid PET/MRI for clinical research and practice.
