# Supplementary material for: A decade of multi-modality PET and MR imaging in abdominal oncology
Source: Br J Radiol. 2021 Jul 22;94(1126):20201351. doi: 10.1259/bjr.20201351 (PMC9328040; doi:10.1259/bjr.20201351)
Supplement: Uncited Supplementary Table 1. [file bjr.20201351.suppl-01.docx]

| **Supplementary Table 1: literature search PubMed query** | | | |
| --- | --- | --- | --- |
|  | | | |
| **Search** | **Items found** | **Search component** | **Search terms** |
| #1 | 189992 | PET | Tomography, Emission-Computed [Mesh] OR petscan* [tiab] OR pet [tiab] OR radionuclid* [tiab] OR (emission [tiab] AND tomograph* [tiab]) |
|  |  |  |  |
| #2 | 738390 | MRI | Magnetic Resonance Imaging [Mesh] OR (magnetic resonance [tiab] AND (image [tiab] OR images [tiab)) OR mri [tiab] OR mris [tiab] OR nmr [tiab] OR mra [tiab] OR mras [tiab] OR mr tomography [tiab] OR mr tomographies [tiab] OR mr tomographic [tiab] OR proton spin [tiab] OR fmri [tiab] OR fmris [tiab] OR zeugmatograph* [tiab] OR ((magneti* [tiab] OR chemical shift [tiab]) AND imaging [tiab]) |
|  |  |  |  |
| #3 | 1486105 | Abdominal Oncology | Abdominal Neoplasms [Mesh] OR Digestive System Neoplasms [Mesh] OR Prostatic Neoplasms [Mesh] OR Urinary Bladder Neoplasms [Mesh] OR Uterine Neoplasms [Mesh] OR Ovarian Neoplasms [Mesh] OR Splenic Neoplasms [Mesh] OR ((Neoplasms [Mesh] OR cancer [tiab] OR cancer* [tiab] OR carcinom* [tiab] OR tumor* [tiab] OR tumour* [tiab] OR neoplas* [tiab] OR metasta* [tiab] OR adenoma* [tiab] OR carcinosarcoma* [tiab] OR hepatoblastoma* [tiab] OR lymphangioma* [tiab] OR lymphangiomyoma* [tiab] OR sarcoma* OR chordoma* [tiab] OR germinoma [tiab] OR gonadoblastoma* [tiab] OR blastoma* [tiab] OR teratoma* [tiab] OR teratocarcinoma* [tiab] OR mesnechymoma* [tiab] OR mesonephroma* [tiab] OR carcinogen* [tiab] OR anticarcinogen* [tiab] OR precancerous [tiab] OR oncolog* [tiab] OR paraneoplastic [tiab] OR precancerous [tiab]) AND abdominal [tiab] OR abdomen [tiab] OR peritoneal [tiab] OR retroperitoneal [tiab] OR digestive [tiab] OR biliary tract [tiab] OR bile duct* [tiab] OR gallbladder [tiab] OR gall bladder [tiab] OR gastrointestinal [tiab] OR esophageal [tiab] OR esophagus [tiab] OR oesophageal [tiab] OR oesophagus [tiab] OR intestinal [tiab] OR intestine* [tiab] OR cecal [tiab] OR cecum [tiab] OR appendix [tiab] OR appendiceal [tiab] OR colorectal [tiab] OR colon [tiab] OR colonic [tiab] OR sigmoid* [tiab] OR rectum [tiab] OR rectal [tiab] OR anus [tiab] OR anal [tiab] OR duodoneal [tiab] OR duodenum [tiab] OR ileal [tiab] OR ileum [tiab] OR jejunal [tiab] OR jejunum [tiab] OR stomach [tiab] OR gastric [tiab] OR liver [tiab] OR hepatic [tiab] OR hepatocellular [tiab] OR pancreatic [tiab] OR pancreas [tiab] OR splenic [tiab] OR spleen [tiab] OR prostatic [tiab] OR prostate [tiab] OR bladder [tiab] OR urothelial [tiab] OR transitional cell* [tiab] OR adnes* [tiab] OR fallopian tumb* [tiab] OR salpin* [tab] OR ovary [tiab] OR ovaries [tiab] OR ovarial [tiab] OR ovarys [tiab] OR ovarian [tiab] OR uterus [tiab] OR uterine [tiab] OR uteral [tiab] OR uteri [tiab] OR cervix [tiab] OR cervical [tiab] OOR myometri* [tiab] OR endometri* [tiab] OR decidu* [tiab] OR gynaecolog* [tiab] OR gynecolog* [tiab] |
|  |  |  |  |
| #4 | 5469 | Combine search #1 and #2 and #3 | #1 AND #2 AND #3 |
|  |  |  |  |
| #5 | 4032 | Dated 2009 - 2018 | AND (2009:2018 [dp] OR 2009:2018 [edat]) |
|  |  |  |  |
| #6 | 3970 | No animals | NOT (Animals [Mesh] NOT Humans [Mesh]) |
|  |  |  |  |
| #7 | 3795 | Original research only | NOT (systematic [sb] OR protocol [ti] OR. Letter [ti] OR review* [ti] OR meta-analys* [ti] OR consensus [ti] OR reply to [ti]) |
|  |  |  |  |
